# Bisdemethoxycurcumin attenuates myocardial fibrosis in heart failure with preserved ejection fraction by targeting TGFBR1 and oxidative stress

**DOI:** 10.1016/j.csbj.2026.01.009

**Published:** 2026-01-14

**Authors:** Rong Xu, Guihua Cao, Liming Hou, Wei Fu, Chenting Bi, Xu Li, Xiaoming Wang

**Affiliations:** Department of Geriatrics, Xijing Hospital, The Airforce Military Medical University, Xi’an, Shaanxi 710032, China

**Keywords:** Bisdemethoxycurcumin, Heart failure with preserved ejection fraction, Network pharmacology, Molecular docking, Experimental validation

## Abstract

Bisdemethoxycurcumin (BDMC), a natural derivative of curcumin with improved solubility and stability, has shown potential cardioprotective properties. This study investigated the efficacy and underlying mechanisms of BDMC in heart failure with preserved ejection fraction (HFpEF) using both *in vivo* and *in vitro* models. The HFpEF mouse model was established using a high-fat diet and L-NAME. BDMC treatment improved cardiac function, attenuated myocardial fibrosis, and exhibited antioxidant effects. Mechanistically, integrated network pharmacology and proteomics identified TGFBR1 as a potential target. BDMC inhibited cardiac fibroblast activation by suppressing TGFBR1 expression and SMAD2/3 phosphorylation. Molecular docking and dynamics simulations confirmed stable binding between BDMC and TGFBR1. These findings demonstrate that BDMC mitigates myocardial fibrosis in HFpEF, primarily by competitively inhibiting the binding of TGF-β and TGFBR1, achieving the effect of inhibiting cardiac fibroblast activation.

## Graphical Abstract

**Figure:**
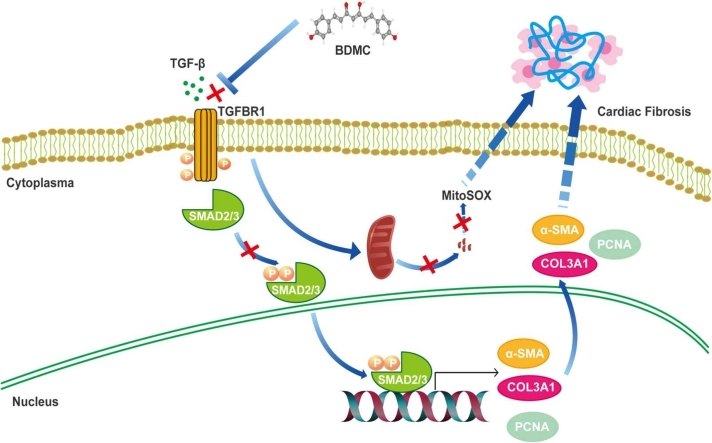

## 1 Introduction

The aging population and rising prevalence of obesity, hypertension, and diabetes have made heart failure (HF) a major global health threat [1]. HF is classified into three subtypes based on left ventricular ejection fraction (LVEF): HF with reduced ejection fraction (HFrEF), HF with mid-range ejection fraction (HFmrEF), and HF with preserved ejection fraction (HFpEF). Its pathophysiology is complex, involving systemic crosstalk between the heart, lungs, kidneys, and other organs, with myocardial fibrosis serving as a key pathological feature driving left ventricular diastolic dysfunction [2], [3]. Despite high morbidity and mortality, therapeutic options for HFpEF remain limited, with SGLT2 inhibitors (e.g., empagliflozin) currently the only evidence-based intervention [4]. This highlights the urgent need to identify novel agents targeting myocardial fibrosis in HFpEF.

The multifactorial risk factors of HFpEF, including obesity, aging, hyperglycemia, and inflammation, necessitate multi-target therapeutic strategies—a gap that natural compounds are well-positioned to bridge due to their low toxicity and pleiotropic effects [5]. Bisdemethoxycurcumin (BDMC), the primary bioactive constituent of turmeric, demonstrates bioactivity superior to that of curcumin, a property attributed to its enhanced hydrophilicity and stability. It possesses anti-inflammatory, anti-oxidative, and metabolic regulatory properties, including suppressing adipogenesis, inhibiting cardiac hypertrophy, and improving hyperglycemia [6], [7], [8], [9]. Our prior work further demonstrated the cardioprotection effect of BDMC against cardiomyocyte injury via the PI3K-AKT-Nrf2/HO1 pathway [10]. However, the efficacy of BDMC in mitigating myocardial fibrosis, the central pathological feature of HFpEF, and its underlying mechanisms have yet to be fully elucidated.

**Fig. 1:**
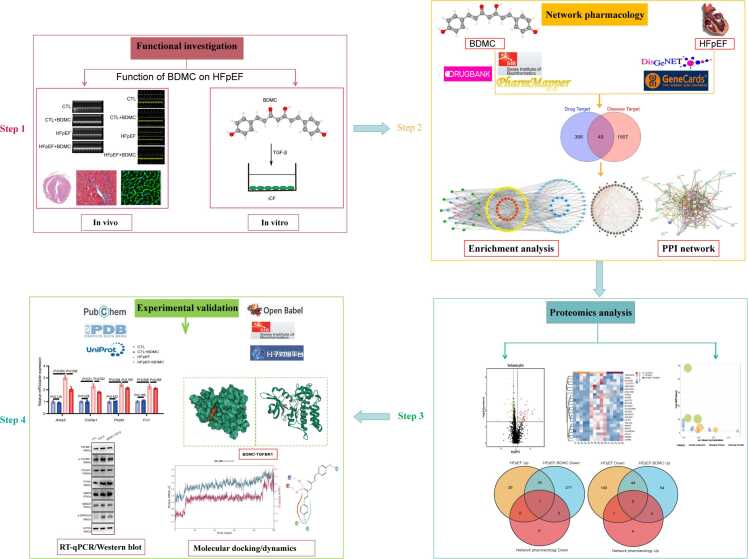
Flow chart of pharmacological targets of BDMC in the treatment of HFpEF based on network pharmacological method and proteomics (BDMC: Bisdemethoxycurcumin, HFpEF: Heart Failure with Preserved Ejection Fraction).

Advances in network pharmacology and bioinformatics have facilitated the identification of natural compounds for complex diseases [11]. Proteomic analysis enables the detection of differential protein expression across groups, specifically the Control, HFpEF, and BDMC-treated HFpEF groups, thereby facilitating the exploration of HFpEF pathogenesis and the mechanisms underlying BDMC's effects. Based on this, the study aimed to evaluate the effects of BDMC on cardiac function, cardiac remodeling and related morphology changes through *in vivo* and *in vitro* models. By integrating network pharmacology and proteomics, our current study was designed to further investigate the therapeutic target of BDMC against HFpEF, and providing a theoretical basis for its clinical application Fig. 1.

## 2 Materials and methods

### 2.1 Chemical and reagents

BDMC (BBP03060) was provided by Yunnan BioBioPha Bio-TechnologyCo. Ltd. (Yunnan, China), and its purity (≥98 %) was confirmed by HPLC (Supplementary 1, Certificate of analysis). 20 mg/kg for BDMC in vivo and 100 μmol L^−1^ for BDMC in vitro (for data see the Support information). TGF-β (HY-P7118) was provided by MCE (Shanghai, China) and 10 ng/mL TGF-β was administered to induce proliferation and activation of immortalized cardiac fibroblast (iCFs).

### 2.2 Animal experiments

Male C57BL/6 J mice (7–8 weeks old) were purchased and bred in a specific-pathogen free environment at the Laboratory Animal Center of the Airforce Military Medical University (AMMU, Xi’an, China). All experimental protocols were approved by the Institutional Animal Care and Use Committee of AMMU (protocol number 20220690), and all procedures were performed in accordance with the Guide for the Care and Use of Laboratory Animals published by the National Institutes of Health guide for the care and use of Laboratory animals (NIH Publications No. 8023, revised 1978).

**Fig. 2:**
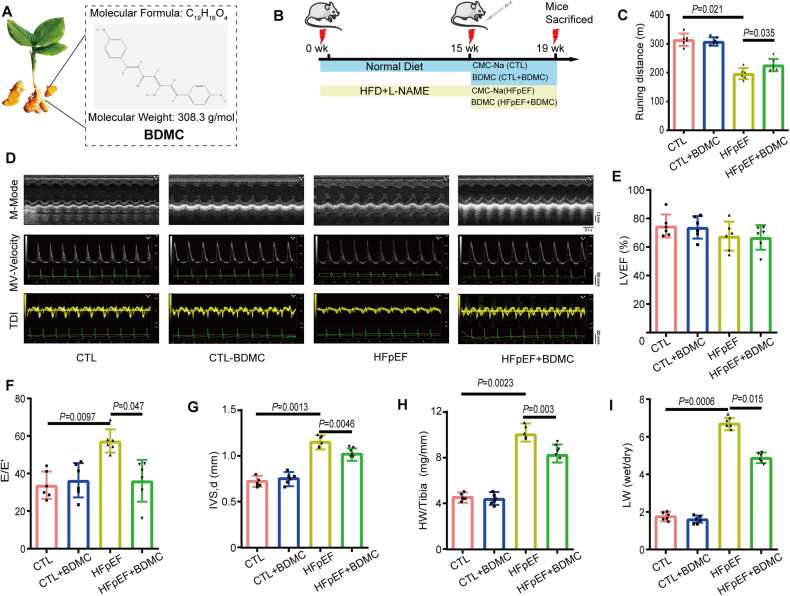
BDMC exerts protective effects on HFpEF *in vivo*. A. Chemical structure of BDMC. B. HFpEF mice was established by administrating high fat diet (HFD) and L-NAME for 16 weeks on male C57BL/6 J mice. BDMC was administered after HFpEF for 4 weeks. C. Running distance of mice after admistrating BDMC. n = 6. D-G. Impact of BDMC on cardiac left ventricular ejection fraction (LVEF) (D, E), E/E’ (F) and Interventricular Septal Diameter (IVS, D) (G). n = 6. **H.** Heart weight to Tibia length ratio (HW/Tibia) of mice after admistrating BDMC. n = 6. I. Lung Weight (LW) wet/dry of mice after admistrating BDMC. n = 6. (CTL:Control group).

The therapeutic effects of BDMC against HFpEF were evaluated using a mouse model established by a high-fat diet (HFD, 60 % kcal from fat, Research Diet) combined with the nitric oxide synthase inhibitor L-NAME (Solarbio, Beijing, China) for 16 weeks [12]. Body weight, lung dry/wet weight, systolic blood pressure, diastolic blood pressure, exercise tolerance and pathophysiological changes of heart were investigated. As shown in Fig. 2, HFD/L-NAME-induced mice displayed an obvious diastolic functional decline, mainly manifested by an increase in the E to E′ ratio. Furthermore, HFD/L-NAME-induced mice exhibited exercise intolerance, an increase in lung weight, cardiac hypertrophy and cardiac fibrosis. Besides, systolic blood pressure (SBP) and diastolic blood pressure (DBP) of HFD/L-NAME-induced mice were also significantly higher than control group. All those characteristics observed are consistent with the symptoms reported in patients with HFpEF [13], [14]. Then control mice and HFpEF mice were randomly divided into two groups and given BDMC (BioBioPha, Yunnan, China) (20 mg/kg) or 0.5 % Sodium carboxymethyl cellulose (CMC-Na) orally for 4 weeks. All mice were anesthetized under isoflurane during surgery and imaging. Body weight, dry/wet weight of lung, exercise tolerance, pathophysiological changes of heart were detected to evaluate the effects of BDMC against HFpEF.

### 2.3 Assessment of cardiac function

Cardiac assessment was performed using transthoracic echocardiography (Vevo 2100, Canada) equipped with an MS-400 imaging transducer, as previously described [15]. Briefly, following the removal of chest hair, mice were anesthetized with 2 % isoflurane via inhalation. A pre-warmed medical ultrasonic couplant was applied to the shaved chest. The probe was initially positioned vertically with the notch oriented towards the mouse’s head. It was then rotated counter-clockwise by approximately 15° to obtain a parasternal long-axis view, ensuring alignment of the apex and outflow tract at the same level. Subsequently, the probe was rotated 90° to acquire a short-axis view, which was recorded for subsequent measurements. Left ventricular ejection fraction (LVEF) was calculated from short-axis M-mode scans using Vevo Lab 3.1.0 software (FUJIFILM VisualSonics, Canada). Echocardiographic data acquisition and measurement were performed independently by two examiners. Cardiac parameters represent the mean of values obtained from three consecutive cardiac cycles, with respiratory artifacts excluded.

### 2.4 Exhaustion test

The exhaustion test was performed after mice were accustomed to treadmill exercise. Procedure of treadmill exercise was set as reported [12], [14]. Briefly, the treadmill was set at a 20° incline. Mice underwent a 4-min warm-up at 5 m/min, followed by 2 min at 14 m/min. Thereafter, the speed was increased by 2 m/min every 2 min until the mouse reached exhaustion. Running time was measured and running distance calculated.

### 2.5 Tail cuff blood pressure recording

The tail blood vessel systolic pressure of awake mice was measured noninvasively on a constant temperature platform (37°C) using the CODA instrument (Kent Scientific, Torrington, CT, USA) as reported [12]. Place the mice on an electric blanket and heat them for 5 min. Put the cuff sensor at the bottom of the tail to record the measurement results. The blood pressure including Systolic Blood Pressure (SBP) and Diastolic Blood Pressure (DBP) of all mice was measured after 3 days of acclimatization to short-term restraint, and was recorded for four consecutive days, 8 measurements were taken each time and the average value was taken.

### 2.6 Tissue collection

Animals anesthetized by isoflurane (RWD, Shenzhen, China) were sacrificed, and their hearts were removed immediately after perfused with 0.9 % saline. For histological analysis, mouse hearts were fixed in 4 % paraformaldehyde (Servicebio, Wuhan, China), embedded in paraffin, and sectioned at 5 μm. Remaining heart tissues were stored at −80°C for subsequent quantitative real-time PCR (RT-qPCR) and western blot analyses.

### 2.7 RNA isolation and Real-time quantitative polymerase chain reaction (qPCR)

RNA isolation and qPCR were carried out as previously described. Briefly, total RNA was extracted according to the manufacturer’s protocol, and cDNA was synthesized using the PrimeScript™ RT Reagent Kit (Takara, Beijing, China). qPCR was performed using TB Green® Fast qPCR Mix (Takara, Beijing, China) on a StepOnePlus Real-Time PCR Detection System (Applied Biosystems, Thermo Fisher Scientific, CA, USA). Relative mRNA expression was calculated using the 2^−ΔΔCt^ method, with β-actin used as the internal control [15]. Primer sequences are listed in Table 1.

**Table 1:** Sequences of PCR primers.

**Gene symbol**	**Accession number**	**Forward primer (5′–3′)**	**Reverse primer (5′–3′)**	**Products size (base pair)**
*Anxa5*	NM_009673	CTTGTGGATGACCTGAAGTCTGAAC	GTAGGCATCGTAGAGTCGTGAGG	90
*Nos3*	NM_008713	CTCTCACCTACTTCCTGGACATCAC	TGCTGTTCGCTGGACTCTTCTG	91
*Igf1r*	NM_010513	GCCAACAAGTTCGTCCACAGAG	GGTAGTAGTCCGTCTCGTAGATGTC	118
*Kdr*	NM_010612	TTCTTCTGGCTCCTTCTTGTCATTG	TGGCATCATAAGGCAAGCGTTC	145
*Tgfbr1*	NM_009370	CGTCGTCCGCAGCTCCTC	TGACTGAGACAAAGCAAAGACCATC	139
*Pim1*	NM_008842	AAGTGGTCCTGTTGAAGAAGGTGAG	GGGCTCCTCGTTCGGTGATAAAG	150
*Esr1*	NM_007956	GCCAAGGAGACTCGCTACTGTG	CAGCCTTCGCAGGACCAGAC	80
*Gsk3B*	NM_019827	ACCACTCAAGAACTGTCAAGTAACC	TGTCCACGGTCTCCAGCATTAG	134
*Dpp4*	NM_010074	CATCATCACCGTGCCAATAGTTCTG	TCTGAAACCCACCACAAAGAGTAGG	132
*Pik3Cg*	NM_020272	CGGGATGCCAAACTCTATGCTATG	GCGGTGGATGACGATGAAGATG	108
*Actin*	NM_007393.5	TGACGGGGTCACCCACACTG	AAGCTGTAGCCGCGCTCGGT	132

### 2.8 Histological analysis

Hematoxylin and eosin (HE) staining, Masson staining and wheat germ agglutinin immunofluorescence (WGA) were conducted as previously described [16]. Heart tissue sections was stained with HE (Servicebio, Wu han, China), Masson (CD069, ZHHC, Xi’an, China) or WGA (Sigma-Aldrich, St. Louis, MO, USA) according to standard protocols. Fibrosis area and WGA-stained regions were quantified using ImageJ software (Wayne Rasband) software.

### 2.9 Cell proliferation assay

Cell proliferation assay was conducted using the 5-ethynyl-2-deoxyuridine (EDU) assay kit (Beyotime, Shanghai, China) following the manufacturer’s instructions. In breif, 1 × 10^4^ iCF per well were seeded in 6-well plate and treating with different plans. Mixing EDU and iCF cell culture media (F12 +10 % FBS) 1:1 and adding the media into iCFs for 2 h. Then iCFs were fixed with 4 % paraformaldehyde solution for 10 min, after successively treating cells with 0.1 % Triton X-100 for 15 min, Click Additive Solution for 30 min, DAPI for 10 min. After washing, images were captured using a Olympus IX73 microscope (Olympus, Germany). The percentage of EdU-positive cells was quantified to show the iCF proliferation.

### 2.10 Therapeutic targets for BDMC against HFpEF

The molecular structure, PubChem CID (5324473) and Canonical SMILES (C1 =CC(=CC=C1/C=C/C(=C/C(=O)/C=C/C2 =CC=C(C=C2)O)/O)O) of BDMC were collected from the PubChem Database (https://pubchem.nc-bi.nlm.nih.gov/). Potential drug targets of BDMC were obtained from PharmMapper Server (http://lilab-ecust.cn/pharmmapper/index.html), Drugbank (https://go.drugbank.com/) and SwissTargetPrediction Databases (http://swisstargetprediction.ch/). Among them, PharmMapper server can identify drug targets using pharmacophore mapping approach [17]. The advantage of DrugBank database is its extensive and experimentally validated drug targets [18]. The SwissTargetPrediction Database mainly predicted potential target candidates based on the similarity of their chemical structures [19]. For SwissTargetPrediction: We specified the probability threshold: targets with a probability > 0.1 were retained. For PharmMapper: We specified the fit score threshold: targets with a normalized fit score ≥ 0.9 were selected. For Drugbank: We specified the relevance score threshold: targets with a relevance score ≥ 10 were included. Drug targets were obtained by merging data from above different databases and deleting the duplicates. Disease targets of HFpEF were obtained from DisGeNET (https://www.disgenet.org/) and Genecards (https://www.genecards.org/). The advantage of DisGeNET lies in its most comprehensive and publicly available collection and its ability to screen targets based on gene-disease correlation scores [20]. Similarly, Genecards database can also calculate the relevance score of diseases and gene targets [21]. These gene names of screening targets were all standardize with the species limited to “Homo Sapiens” in Uniprot (https://www.uniprot.org/). After merging targets from different databases and deleting the duplicates, therapeutic targets for BDMC against HFpEF were identified by intersecting the drug targets and disease targets.

### 2.11 Network construction of diseases‑targets‑functional annotations‑signaling pathways for BDMC against HFpEF

The protein-protein interaction (PPI network) of the intersection targets was analyzed using the Search Tool for Retrieval of Interacting Genes/Proteins (https://www.string-db.org/) with a confidence score > 0.40 and GeneMANIA (http://genemania.org/). Enrichment analyses of Gene Ontology (GO) functional annotation and Kyoto Encyclopedia of Genes and Genomes (KEGG) pathway and disease association analysis were performed by using the DAVID database (https://david.ncifcrf.gov/) and visualized by Cytoscape software. The top 20 enriched GO annotations including molecular function, biological process and cellular component, and KEGG terms with P < 0.05 were selected and then disease-target-functional annotation-signaling pathway network was finally visualized in Cytoscape software. Subcellular localization of candidate target molecules were obtained using the UniProt database (www.uniprot.org), membrane proteins were prioritized for molecular docking with BDMC.

### 2.12 Molecular docking validation of the binding capacity between BDMC and targets

The X-ray crystal structure of candidate target molecules was retrieved from the Protein Data Bank (http://www.rcsb.org/pdb/home/home.do). The PubChem CID of BDMC were collected from the PubChem Database (https://pubchem.nc-bi.nlm.nih.gov/). For docking analysis, all protein and molecular files were converted into PDBQT format with all water molecules excluded and polar hydrogen atoms were added. The grid box was centered to cover the domain of each protein and to accommodate free molecular movement.

The methodological details for the docking: Docking Grid (Binding Site Definition): The grid box was centered on the active site with the following coordinates and dimensions: Center: X = 10.729 Å, Y = -0.714 Å, Z = 12.09 Å. Size: X = 48.51 Å, Y = 66.87 Å, Z = 79.98 Å. This box size was chosen to be sufficiently large to fully accommodate the ligand and allow for flexible movement during docking. Force Field: The OPLS2005 force field was used for both the protein and the ligand. Scoring Function: The standard Glide scoring function (GlideScore) was employed for pose ranking and selection, as implemented within the Schrödinger Suite. No non-default settings were applied to the scoring function.

The binding capacity of targets with BDMC was assessed by molecular docking using Docking Web Server (GRAMM) (https://www.dockeasy.cn). The smaller binding energy means it is easier to bind with BDMC. So the first 10 targets according to the binding energy were chosen for further validation.

### 2.13 Label-free quantitative proteomics

Heart samples from Control (Normal diet) mice (n = 5), HFpEF mice (n = 5) and BDMC-treated HFpEF mice (n = 5) were used for label-free quantitative proteomics assays. Experiments were performed as previously described [22]. Briefly, heart samples were cut into pieces and lysed in RIPA buffer supplemented with protease and phosphatase inhibitor cocktails (Beyotime Biotechnology, China). Then, samples were homogenized on ice using a Bullet Blender Tissue Homogenizer (Next Advance, USA). After homogenization, samples were centrifuged at 12, 000 g for 20 min at 4 °C, and protein concentrations of the supernatant were determined by the Bradford assay. After overnight trypsin digestion supplemented with 50 μL of 50 mM NH_4_HCO_3_, the resulting peptides were analyzed using an Orbitrap Fusion™ Lumos™ Tribrid™ mass spectrometer (Thermo Fisher Scientific, USA) coupled online to an Easy-nLC 1000 system. Peptides were loaded onto a trap column (Thermo Fisher Scientific, USA) and subsequently separated on an analytical column (Thermo Fisher Scientific, USA) using an acetonitrile gradient. Mass spectrometry (MS) data were acquired in positive-ion mode with an electrospray voltage of 2300 V. Full MS scans were performed over the range of 350–1500 *m/z* at a resolution of 60,000. The automatic gain control (AGC) target was set to 4e5, with a maximum injection time of 50 ms. The MS cycle time was 3 s, utilizing data-dependent acquisition (DDA) with automated precursor peak selection. Precursor ions were selected for fragmentation based on the following criteria: highest intensity peaks, ion intensity threshold of 5e3 counts, and charge states of 2 + to 7 + . MS/MS spectra were acquired for the selected precursors. Raw MS data files were analyzed using Proteome Discoverer™ software (version 2.4, Thermo Fisher Scientific, USA). Database searches were performed against the UniProtKB mouse protein database (www.uniprot.org) for protein identification. Principal component analysis (PCA), volcano analysis, and GO annotation enrichment analysis were performed to explore the mechanism of BDMC against HFpEF. Differential protein analysis involves screening for proteins with significantly altered abundance between comparative groups based on quantitative proteomics results. The analysis first performed Student’s T-test using R to obtain p-values for protein abundance in each comparison group. For samples with biological replicates, proteins with significant differences between groups were identified based on the following thresholds: absolute fold change (FC) > 1.5 and p-value < 0.05.

Clustering analysis is often used to reflect the expression patterns of differentially expressed proteins under different experimental conditions. Functionally related proteins usually exhibit similar expression patterns under the same conditions—for example, proteins regulated by common transcription factors or involved in the same biological processes. Hierarchical clustering analysis was performed on differentially expressed proteins from the comparison groups. For experiments with more than two samples, Z-score normalization was applied before plotting. In the heatmap, each column represents a sample, each row represents a protein, and the expression level of a protein across samples is indicated by color intensity: red indicates higher expression, while green indicates lower expression. This clustering was applied specifically to the differentially expressed proteins and did not involve specifying distance metrics, linkage methods, or cluster cutoffs.

### 2.14 Western blotting analysis

Western blotting analysis were performed using standard protocols [16]. Primary antibodies against the following proteins were used: TGFBR1 (abs11370, Absin, China), p-TGFBR1 (abs139909, Absin, China), Col3A1 (abclonal A0817, China), PCNA (GB11010, Servicebio, Wuhan, China), MMP9 (GB12132, Servicebio, Wuhan, China), SMAD3 (66516, Proteintech, Wuhan, China), p-SMAD2/3 (80427, Proteintech, Wuhan, China), MFN1 (TD7543, Abmart, China), MFN2 (T56638, Abmart, China), DRP1 (TD7037, Abmart, China), TOMM20 (T55527, Abmart, China), β-ACTIN (AT0001, Engibody, Beijing, China). The membranes were visualized via chemiluminescence using ChemiDoc XRS + imaging system (Bio-Rad, USA). The relative band densities in all western blot images were quantified with ImageJ software (Wayne Rasband) and analyzed using Graphpad Prism 10 (La Jolla, CA).

### 2.15 Immunofluorescence Staining

Cells grown on coverslips were fixed with 4 % paraformaldehyde for 15 min at room temperature, followed by permeabilization with 0.1 % Triton X-100 for 10 min. After blocking with 5 % bovine serum albumin (BSA) for 1 h, samples were incubated overnight at 4°C with primary antibodies against TGF-β (GB115739, Servicebio, China) and TGFBR1 (GB14152, Servicebio, China). Subsequently, samples were incubated with appropriate fluorophore-conjugated secondary antibodies (GB25303, GB28303, Servicebio, China) for 1 h at room temperature in the dark. Cell nuclei were counterstained with DAPI. Images were captured using a confocal microscope.

### 2.16 Molecular dynamics simulations

The molecular dynamics simulations were performed using the academic version of Desmond/Maestro 2022.1 [23], [24]. Each system was solvated with TIP3P water molecules and neutralized with 0.15 M NaCl. Following energy minimization and equilibration, the production simulation was carried out for 100 ns under isothermal-isobaric conditions (300 K, 1 bar). Trajectory frames were saved at intervals of 100 ps for subsequent analysis. All analyses were performed using the Simulation Interaction Diagram tool integrated in Desmond.

The methodological details for molecular dynamics simulations: System Setup: The solvated system was placed in an orthorhombic simulation box. Simulation Box Dimensions: The final box volume was approximately 361,708 Å3. Force Field: The OPLS2005 force field was consistently used throughout the MD simulations. Temperature Control: Temperature was maintained at 310 K using the Nosé-Hoover Chain thermostat. This algorithm is effective at generating a correct canonical ensemble and minimizing temperature oscillations. Pressure Control: Pressure was maintained at 1.01325 bar (1 atm) using the Berendsen barostat. We acknowledge that while this barostat efficiently equilibrates the system pressure, it does not rigorously produce an isothermal-isobaric (NPT) ensemble. Its use here was for the initial equilibration phase to achieve stable density rapidly.

### 2.17 ROS detection

ROS production was detected using Reactive Oxygen Species (ROS) Fluorometric Assay Kit (Elabscience, Shanghai, China) following the manufacturer’s instructions. For in vitro analysis, post-treatment cells from each group were incubated with 10 μM DCFH-DA, which can emit green fluorescence in the presence of ROS. The fluorescent signal was then read using microplate reader (H1, Biotek, USA) at 500/530 nm.

### 2.18 Transmission electron microscopy (TEM)

Small cubic pieces ≤ 1 mm^3^ from left ventricles were fixed in a solution containing 4 % paraformaldehyde and 0.3 % glutaraldehyde, followed by thorough washing, graded acetone dehydration, and embedding in LR White resin (Sigma-Aldrich). Ultrathin sections (50–70 nm) were mounted on nickel grids and sequentially stained with uranyl acetate and lead citrate. Imaging was performed using an EM 109 R electron microscope (Zeiss, Germany).

### 2.19 Mitochondrial structure analysis

To assess changes in mitochondrial structure, cells were stained with MitoTracker® Red CMXRos ((Yeasen, Shanghai, China) at 1 mM for 10 min at 37 ℃. Confocal microscope was used to capture single-cell images assisted by the Image-J software to acquire mitochondrial length.

### 2.20 Mitochondrial superoxide assay

Mitochondrial superoxide assay was conducted using MitoSOX Red GR-30921 assay kit (Yeasen, Shanghai, China) following the manufacturer’s instructions. In brief, iCF cells (1 ×10^4^ cells/well) were cultured in 6-well plates and treating with different plans. Then iCFs were washed with PBS and trearted with mitoSOX red (2 µM) for 30 min, DAPI for 10 min. After washing, images were captured using a Olympus IX73 microscope (Olympus, Germany). The percentage of MitoSOX-positive cells was quantified to show the mitochondrial superoxide production of iCF.

### 2.21 ATP assay

ATP assay was conducted using ATP chemiluminescence assay kit (Elabscience, Shanghai, China) following the manufacturer’s instructions. In brief, The lysed cells from each group were mixed with Reagent I and immersed in boiling water for 10 min, then was centrifuged for 10 min at 4 °C, 10,000 × g, the supernatant was collected in a 96-well, black opaque plate. The supernatant of sample/standard was mixed with freshly prepared Working Reagent and incubated at room temperature for 5 min, then read using microplate reader (H1, Biotek, USA) within 30 min. The sample concentration of ATP was calculated according to an ATP standard curve.

### 2.22 Statistical analysis

All data were analyzed using GraphPad Prism 10 software and are presented as the mean ± standard deviation (SD). Statistical significance were analyzed using Student’s *t*-tests, multiple *t*-tests, or one-way ANOVA. *P* < 0.05 was set as the threshold for statistical significance. Data analysis was carried out by researchers who were unaware of the experimental design.

## 3 Results

### 3.1 BDMC improves cardiac function and reduces myocardial fibrosis in HFpEF mice

To investigate the role of BDMC in HFpEF, we analyzed the effects of BDMC in a HFpEF mouse model. After the establishment of HFpEF, Control mice (Normal diet) and HFpEF mice (HFD+L-NAME) were randomly divided into two groups and given BDMC (20 mg/kg) or 0.5 % CMC-Na orally for 4 weeks, respectively (Fig. 2B). The administration of BDMC resulted in a significant improvement in running distance (Fig. 2C). Echocardiographic analysis revealed that the administration of BDMC (but not CMC-Na) attenuated HFpEF-induced increases in the E/E’ ratio, Interventricular Septal Diameter (IVS,d), heart weight/tibia (HW/Tibia), and Lung Weight (LW) wet/dry, without affecting ejection fraction (Fig. 2D-I). Echocardiographic analysis of E/A ratio was shown in Supplementary Figure S1A, S1B.

**Fig. 3:**
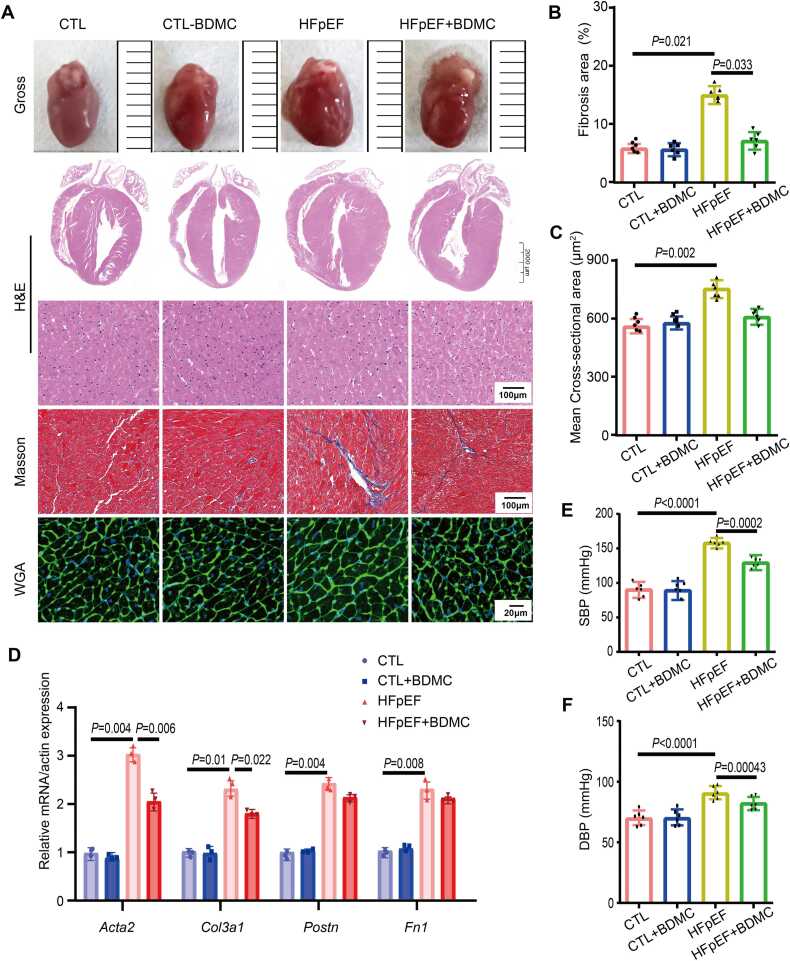
BDMC exerts anti-fibrotic effect *in vivo*. A. Representative gross images of whole hearts, staining with H&E, Masson, wheat germ agglutinin (WGA) of mice after admistrating BDMC. B. Quantification of fibrosis area percent of different group. n = 6. C. Mean cross-sectional area of different group. n = 6. D. qPCR analysis of fibrosis-related genes *Acta2, Col3a1, Postn, Fn1 in vivo* in the indicated groups. E-F. Systolic Blood Pressure (SBP) (E) and Diastolic Blood Pressure (DBP) (F) of mice after admistrating BDMC. n = 6. (CTL:Control group).

Furthermore, Masson staining was performed to evaluate cardiac fibrosis and wheat germ agglutinin (WGA) staining was used to determine cardiac hypertrophy by measuring the mean cross-sectional area of cardiomyocytes. As shown in Fig. 3A-C, Masson staining revealed that BDMC mitigates cardiac fibrosis significantly (Fig. 3B), while the mean cross-sectional area of cells did not change significantly (Fig. 3C). Additionally, the transcription of fibrosis-related genes was decreased after BDMC treatment (Fig. 3D). Moreover, SBP and DBP were increased in HFpEF and were decreased after the administration of BDMC (Figs. 3I, 3J). These findings suggest that BDMC exhibits a protective effect against HFpEF in vivo.

### 3.2 BDMC inhibits activation and proliferation of cardiac fibroblasts in vitro

**Fig. 4:**
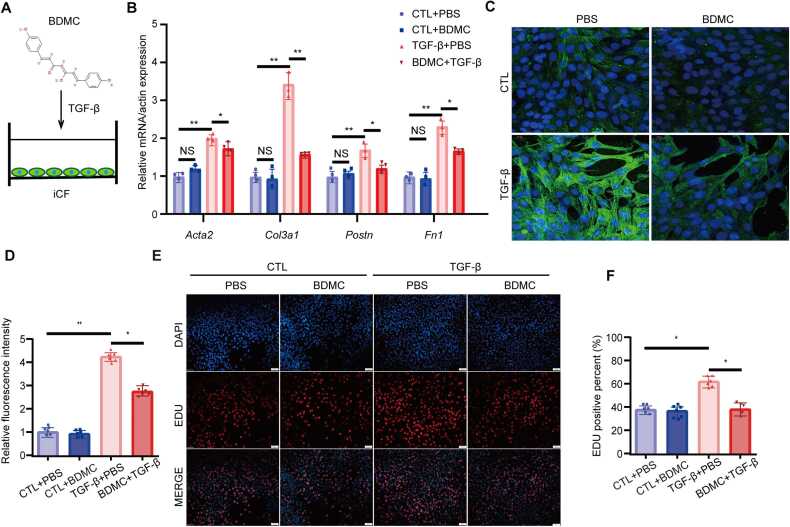
BDMC exerts anti-fibrotic effect in iCFs *in vitro*. A. Impact of BDMC on TGF-β induced stimulation of immortalized cardiac fibroblasts (iCF) was tested in vitro. B. qPCR analysis of fibrosis-related genes Acta2, Col3a1, Postn, Fn1 in vitro in the indicated groups. C. Representative images of α-smooth muscle actin (α-SMA) immunofluorescence of iCF in the indicated groups. D. Expression of α-smooth muscle actin (α-SMA) of iCF in the indicated groups. n = 6. E. Representative images of EDU assay of iCF in the indicated groups. F. Quantification of EDU positive percent in the indicated groups. (CTL:Control group).

Given the protective effects of BDMC against HFpEF *in vivo*, we next assessed its anti-fibrotic and antioxidant properties *in vitro*. 10 ng/mL TGF-β (MCE, Shanghai, China) treated iCFs (Fig. 4A) were used to assess the impact of BDMC on fibrosis and oxidative properties. BDMC attenuated iCFs activation, indicated by the decreased mRNA of fibrosis-related genes *Acta2, Col3a1, Postn, Fn1 in vivo* and *in vitro* (Figs. 3D, 4B) and a reduction in α-smooth muscle actin (α-SMA) expression (Figs. 4C, 4D). Furthermore, as measured by EDU staining, proliferation of iCFs induced by TGF-β was decreased significantly after treatment with BDMC (Figs. 4E, 4F).

### 3.3 Network pharmacological screening and analysis of therapeutic targets for BDMC against HFpEF

**Fig. 5:**
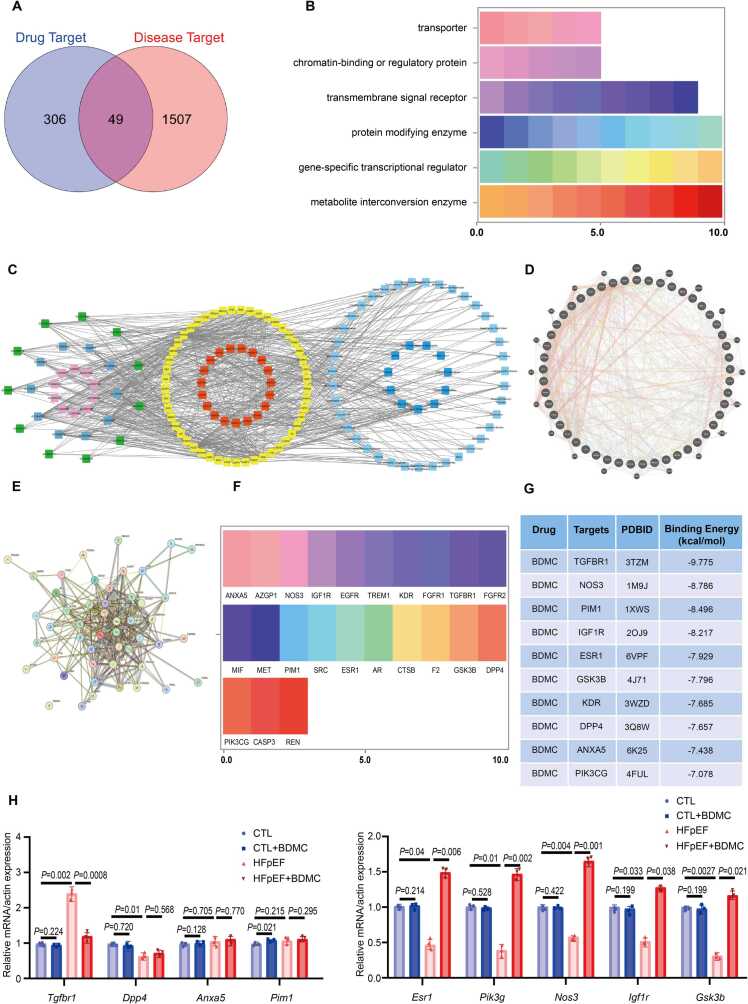
Identification of therapeutic targets for BDMC against HFpEF. A.Venn diagram of potential therapeutic targets. B. Classification of the identified targets. 49 therapeutic targets for BDMC against HFpEF can be obtained by the intersection of 3066 drug targets and 1507 HFPEF-related targets and classified into 6 functional categories. C. Diseases-targets-functional annotations-signaling pathways network. GO analysis results are on the left. The dark green quadrangles represent cellular components. The blue quadrilateral represents biological processes. The pink quadrilateral represents the function of the molecule. The yellow circle in the middle indicates targets for BDMC against HFpEF. The central red quadrilateral represents the enriched KEGG pathway. Disease enrichment analysis is represented by the blue diamond on the right. (*GO: Gene ontology, KEGG: Kyoto Encyclopedia of Genes Genomics*). D. mRNA interaction network of therapeutic targets for BDMC against HFpEF. E. Protein–protein interaction network of therapeutic targets for BDMC against HFpEF. F. Target molecules located on the cell membrane. G. Molecular docking binding energy of BDMC with target proteins. **H.** Relative mRNA expression of targets in different treatment groups of mice. n = 3. (CTL:Control group).

To investigate the key therapeutic targets for BDMC against HFpEF, network pharmacological screening was conducted. As shown in Fig. 5A, there are 101 and 266 BDMC drug targets in Drugbank and SwissTargetPrediction databases, respectively. These drug targets were combined and 61 duplicates were removed. Finally, 306 drug targets were selected for further analysis. Similarly, there were 1516 and 48 targets associated with HFpEF in the DisGeNET and Genecards databases, respectively. After removing redundant targets, 1507 HFpEF-associated disease targets were identified for further analysis.

By taking the intersection of the above BDMC drug targets and HFpEF targets, 49 potential therapeutic targets of BDMC against HFpEF were obtained (Fig. 5A). These targets can be divided into 6 functional categories: transporters (ANXA5, TTR, AZGP1, ALB, NOS3), chromatin-binding or regulatory protein (BCHE, YARS1, TNNC1, MIF, CCNA2), transmembrane signal receptor (IGF1R, EGFR, TREM1, KDR, FGFR1, TGFBR1, FGFR2, ESRRG, MET), protein modifying enzyme (MAPK8, MAPK14, PTPN11, PDE5A, YARS1, PIM1, SRC, CDK2, MMP8, ESR1), gene-specific transcriptional regulator (NR1H2, AR, SHBG, PPARG, PPIA, PGR, PARP1, HSP90AA1, CTSK, F2) and metabolite interconversion enzyme (ALDH2, GSTP1, GSK3B, MTHFD1, DPP4, PIK3CG, CASP3, REN, GSR, CASP7) (Fig. 5B).

Disease-target-functional annotation-signaling pathway network analysis was performed for these 49 therapeutic targets for BDMC against HFpEF. GO, KEGG and disease enrichment analysis were performed in the DAVID database. The results presented a total of 11 cell components and the top 20 enrichment of the biological processes, molecular functions and KEGG pathways in Fig. 5C (Fig. 5C). The BP results showed higher enrichment in apoptotic process, epithelial to mesenchymal transition, cellular response to oxidative stress, and negtive regulation of SMAD protein import into nucleus. The CC for BDMC against HFpEF mainly involved cytosol, cytoplasm, plasma membrane, receptor complex, ficolin-1-rich granule lumen were mainly concentrated. The protein serine/threonine/tyrosine kinase activity, enzyme binding, RNA polymerase II transcription factor activity, transmembrane receptor protein tyrosine kinase activity, and protein tyrosine kinase activity were the main enrichment in MF. KEGG pathway enrichment results showed that PI3K-Akt signaling pathway, MAPK signaling pathway, diabetic cardiomyopathy, Rap1 signaling pathway and adherens junction were significantly enriched. The GO and KEGG analysis results indicated the complex and multifarious impacts of BDMC on HFpEF (Fig. 4C). Besides, a Genemania network based mRNA (Fig. 5D) and a protein-protein interaction (PPI) network (Fig. 5E) were constructed using 49 identified therapeutic targets for BDMC against HFpEF. The PPI network consists of 49 nodes and 316 edges, with an average node degree of 12.9 (Fig. 5E).

Given that cellular entry via the plasma membrane is a prerequisite for BDMC's activity, the initial target screening was based on subcellular localization. Using the UniProt database (www.uniprot.org), we found that 23 out of the 49 candidate target molecules are annotated to be located in the cell membrane (Fig. 5F). These 23 membrane proteins were therefore prioritized for molecular docking with BDMC. Smaller binding energy indicates stronger binding affinity to BDMC. So the first 10 targets according to the binding energy were chosen for further validation (Fig. 5G). Cardiac tissues of different group of mice were collected, RT-qPCR was performed to verify the mRNA expression of 10 molecules. The sequences of the primers used for RT-qPCR are shown in Supplementary Table 1. According to the RT-qPCR results in Fig. 5H, *Tgfbr1* was significantly up-regulated in HFpEF and significantly down-regulated after BDMC treatment. *Esr1, Pik3cg, Nos3, Igf1r and Gsk3b* were significantly down-regulated in HFpEF and significantly up-regulated after BDMC treatment. *Kdr* was not detected in the indicated groups (Fig. 5H).

### 3.4 Integrative proteomics and network pharmacology confirm TGFBR1 as a potential anti-fibrotic target

**Fig. 6:**
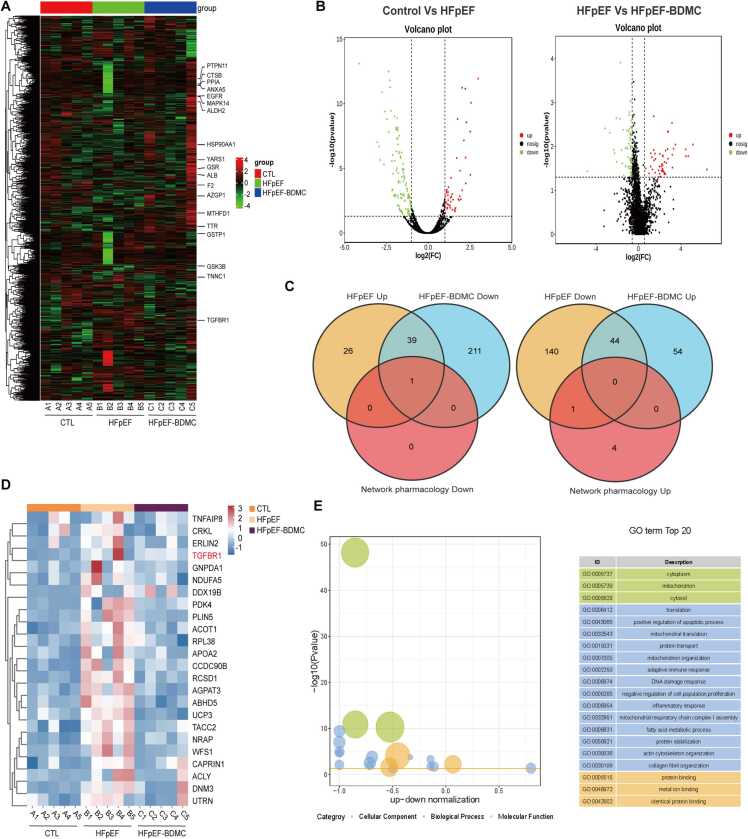
Integrating proteomics analysis and network pharmacology analysis of BDMC against HFpEF. A. Heatmap of all the protein expression in the three groups. B. Volcano plots of the enriched proteins. C. Venn diagram of the integrated proteins of proteomics analysis and network pharmacology analysis. D. Heatmap of the enriched different expression proteins of up-regulated in HFpEF and down-regulated in HFpEF-BDMC compared with HFpEF. E. Enriched differential bubble chart of proteins in Figure D.

Based on the results above, we obtained 6 pivotal targets of BDMC against HFpEF by network pharmacology analysis. Furthermore, label-free quantitative proteomic analysis was used to identify differentially expressed proteins in the ventricle of the Control group, HFpEF group and HFpEF-BDMC group. As shown in Fig. 6A, 2690 proteins were detected in the three groups (Fig. 6A). To screen for differentially expressed proteins in the indicated groups, we used the screening standards with 1.5-fold change differences and *P* value < 0.05 to select the probable targets. All of 251 targets were significantly differential expression at the protein level between HFpEF and Control group, and 349 targets were found between HFpEF-BDMC and HFpEF group, as visualized by volcano plot (Fig. 6B). To better investigate the target of BDMC against HFpEF, we integrated the proteomics analysis and the 6 targets (1 up-regulated target and 5 down-regulated targets) screened by network pharmacology analysis. Different protein expression analysis of the proteomics revealed that one target was up-regulated in HFpEF compared with Control while down-regulated in HFpEF-BDMC compared with HFpEF (TGFBR1). However, NOS3 was down-regulated in HFpEF compared with Control while they did not change significantly in HFpEF-BDMC compared with HFpEF (Fig. 6C). Because the HFpEF mouse model was established by applying HFD and L-NAME, L-NAME was the inhibitor of NOS, which is consistent with the change of NOS3. Furthermore, hierarchical clustering analysis of the indicated groups showed that TGFBR1 was the target up-regulated in HFpEF and down-regulated in HFpEF-BDMC compared with HFpEF (Fig. 6D). The enriched differential bubble chart of these different expression proteins in Fig. 6D was presented in Fig. 6E. It also indicated mitochodrial-related oxidative stress and fibrosis significantly changed after administration of BDMC (Fig. 6E). Collectively, TGFBR1 was identified as the potential therapetic target of BDMC against HFpEF.

### 3.5 BDMC inhibits the TGFBR1-SMAD2/3 pathway to suppress myocardial fibrosis

Network pharmacology and proteomics data showed that the core protein TGFBR1 may play an important role in the treatment of BDMC against HFpEF. As mentioned previously, iCFs activation induced by TGF-β leads to fibrosis, which is one of the main pathological processes of HFpEF [25]. Moreover, activation of the TGF-β/SMAD pathway resulted in fibrosis leads to left ventricular diastolic dysfunction [26]. Therefore, the expression levels of SMAD pathway-related fibrotic proteins in BDMC treated HFpEF mice and the process of TGF-β induced iCFs activation were explored.

**Fig. 7:**
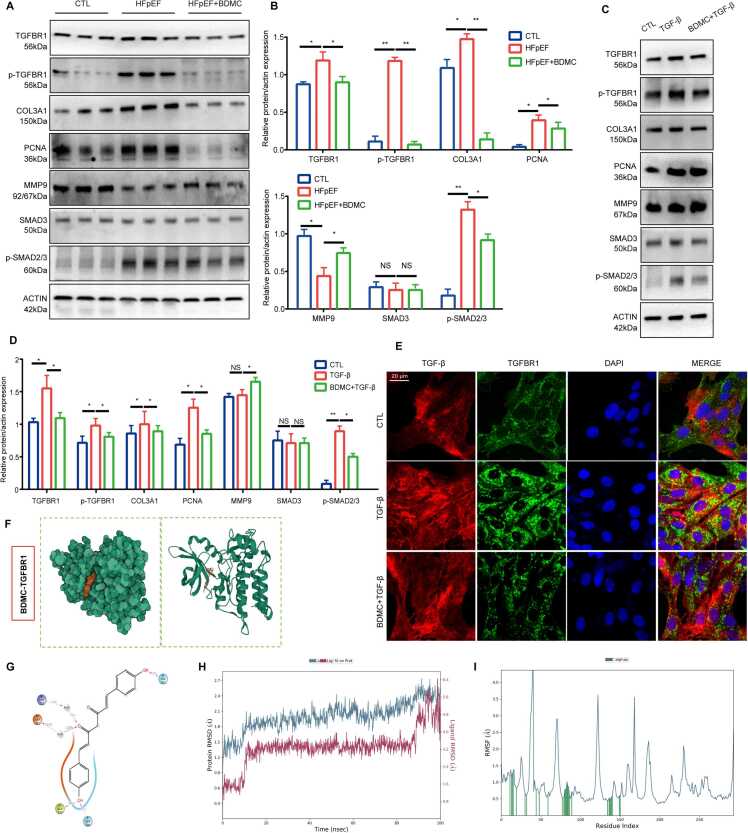
Expression of TGF-β/SMAD pathway-related fibrotic proteins in BDMC treated HFpEF. A. Protein bands of TGFBR1, p-TGFBR1, COL3A1, PCNA, MMP9, SMAD3, p-SMAD2/3 in the indicated groups. B. Relative protein expression of targets in different treatment groups of mice. n = 3. C. Protein bands of TGFBR1, p-TGFBR1, COL3A1, PCNA, MMP9, SMAD3, p-SMAD2/3 in the indicated groups. D. Relative protein expression of targets in different treatment groups of mice. n = 3. E. Immunofluorescence Staining of TGF-β, TGFBR1, DAPI in the indicated groups. F. The 3-dimensional map of the binding sites between BDMC with TGFBR1. G. Molecular dynamics (MD) simulation of BDMC binding to TGFBR1. H. Root Mean Square Deviation (RMSD) of BDMC and TGFBR1. **I.** Root Mean Square Fluctuation (RMSF) of BDMC and TGFBR1.

As shown in Fig. 7 A and B, compared with the control mice, the expression of TGFBR1 and phosphorylation levels of TGFBR1 and SMAD2/3 were increased in HFpEF mice and significanly decreased after BDMC treatment. Consistently, the fibrosis-related proteins COL3A1 and PCNA were also increased in HFpEF, decreased after treating mice with BDMC. In iCFs, the elevated expression levels of TGFBR1, phosphorylation levels of TGFBR1, SMAD2/3, and the fibrosis-related proteins COL3A1 and PCNA in TGF-β-treated iCFs were confirmed by Western blotting (Fig. 7 C and 7D). This finding suggested that BDMC can play an anti-fibrotic role by inhibiting the TGF-β/SMAD pathway-related proteins during HFpEF. Immunofluorescence Staining in Fig. 7E showed an increased expression of membrane-located TGFBR1 after cells were treated with TGF-β, but it decreased after pretreated cells with BDMC (Fig. 7E).

Further molecular docking was performed and shown in Fig. 7 F. The docking models with the lowest binding energy between BDMC and mutiple potential targets were discussed above and presented in Fig. 5G. The results showed that BDMC exhibited the strongest and most stable binding affinity toward TGFBR1, followed by NOS3, IGF1R, ESR1, GSK3B and PIK3CG.

After docking, the accuracy of the ligand protein complex obtained by scoring function is not very high. At this stage, molecular dynamics simulation is required to investigate the interaction mechanism between the drug and target protein, providing direct insights for drug design and the discovery of new compounds. As shown in Fig. 7H, the Root Mean Square Deviation (RMSD) of the protein remained in a relatively stable state in the simulation process, and finally stabilized between 1.8 and 2.4 A, while the final RMSD of the ligand stabilized around 4.8 A after fluctuations, indicating that the initial conformation of BDMC and TGFBR1 is relatively stable, and after dynamic simulation, a more stable combined conformation is formed on the original basis. The Root Mean Square Fluctuation (RMSF) values of combined amino acid residues between BDMC and TGFBR1 (marked in green line in Fig. 7I) also confirmed the stable combined conformation of BDMC and TGFBR1. Additionally, multiple forces were formed between BDMC and TGFBR1, including the water bridge of the amino acid ASP351 and other hydrogen bonds showed in Fig. 7 G.

### 3.6 Inhibition of mitochondrial-related oxidative stress contributed to the effects of BDMC against HFpEF

**Fig. 8:**
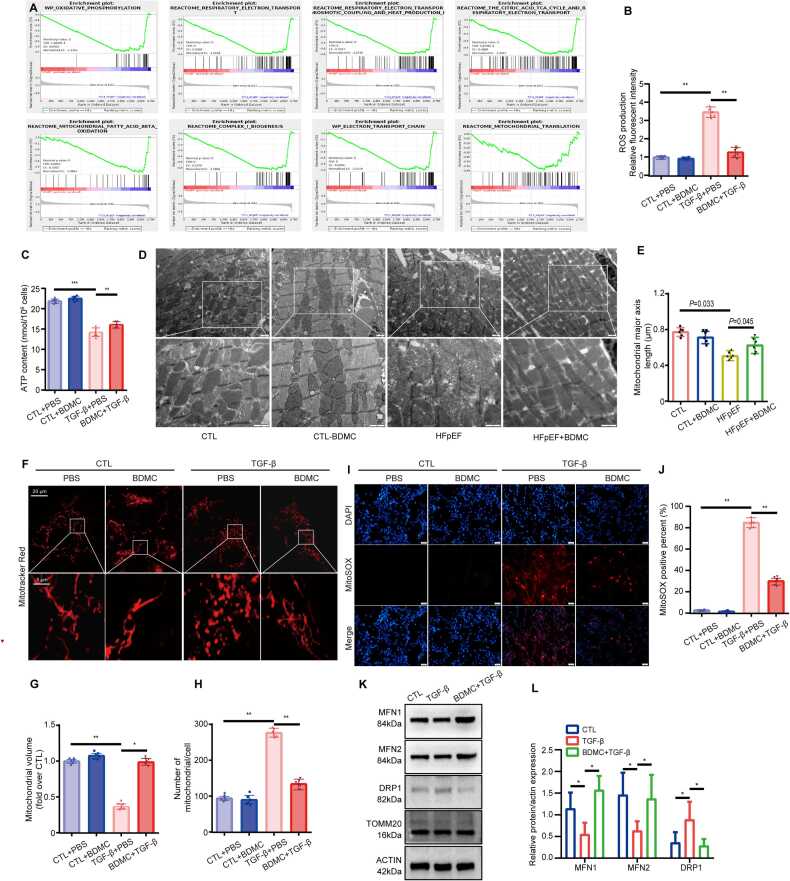
BDMC inhibited mitochondrial-related oxidative stress *in vivo* and *in vitro*. A. Gene Set Enrichment Analysis (GSEA) of HFpEF-BDMC (indicated PC4-HFpEF). B. ROS production was quantified by DCFH-DA in vitro. n = 6. C. ATP content in the indicated groups. n = 6. D. Representative transmission electron microscopy (TEM) images. n = 6. E. Mitochondrial length (major axis) in the indicated groups. n = 6. F. Representative mitochondrial immunofluorescence using Mitotracker (red). n = 6. G. Mitochondrial volume (fold over CTL) in the indicated groups. n = 6. H. Number of mitochondrial/cell in the indicated groups. n = 6. I. Representative images of mitoSOX assay of iCF in the indicated groups. n = 6. J. Quantification of mitoSOX positive percent in the indicated groups. n = 6. K. Representative protein bands of MFN1, MFN2, DRP1, TOMM20 and ACTIN in the indicated groups. **L.** Relative protein expression of targets in different treatment groups. n = 3.

As showed in Fig. 6E, mitochodrial-related oxidative stress and fibrosis-related proteins were significantly enriched after BDMC administration. Since mitochondrial dysfunction in the heart is a hallmark of heart failure and a major source of oxidative stress. Oxidative stress, in turn, has an adverse effect on cellular components, including mitochondria themselves, thus creating a vicious cycle. Oxidative stress can also lead to myocardial tissue damage and inflammation, thereby causing the progression of heart failure. Whether BDMC protected heart by inhibiting mitochondrial function, the marker of mitochondrial function, ATP synthesis, production of ROS and mitoSOX were studied. As shown in Fig. 8A, Gene Set Enrichment Analysis (GSEA) revealed that oxidative phosphorylation, respiratory electron transport, the tricarboxylic acid (TCA) cycle, mitochondrial fatty acid β-oxidation, complex I biogenesis, the electron transport chain and mitochondrial translation were all negatively correlated with HFpEF-BDMC (indicated PC4-HFpEF in Fig. 8A). The inhibition of ATP synthesis induced by TGF-β can be relieved by applying of BDMC, while exceed production of ROS was inhibited by BDMC (Figs. 8B, 8C). Furthermore, mitochondrial ultrastructure obtained by transmission electron microscopy (TEM) showed decreased mitochondrial major axis length in HFpEF mouse hearts, while relatively longer mitochondrial major axis length in BDMC treated HFpEF (Fig. 8D-E). *In vitro*, TGF-β significantly increased the mitochondrial fragmentation (Fig. 8F-H), ROS production (Fig. 8B) and mitoSOX levels (Fig. 8I-J), all of which were inhibited by BDMC (Fig. 8D-I). In addition, the expression of mitochondria-related regulatory proteins was assessed (Fig. 8K). TGF‑β‑treated iCFs exhibited reduced levels of the mitochondrial fusion proteins MFN1 and MFN2, which were restored by pretreatment with BDMC. In contrast, the mitochondrial fission protein DRP1 displayed an opposite expression pattern. Notably, TOMM20 expression remained largely unchanged across all groups. These results collectively indicate that BDMC exerts anti-oxidative stress effects partially via moduating mitochondrial dynamics.

## 4 Discussion

The escalating global prevalence of aging populations, obesity, hypertension, and diabetes has positioned heart failure with preserved ejection fraction (HFpEF) as a critical cardiovascular challenge, characterized by limited therapeutic options and substantial socioeconomic burden [1]. Bisdemethoxycurcumin (BDMC), a natural bioactive compound derived from Curcuma longa L., demonstrates multifaceted therapeutic potential, including anti-obesity (suppressing adipogenesis), anti-hypertrophy (p300-HAT activity modulation), anti-hyperglycemia (inhibiting pancreatic α-amylase) and improving metabolic disorders such as hyperinsulinemia, hyperlipidemia, and inflammation. Moreover, BDMC exhibits enhanced solubility and bioavailability compared to other curcuminoids, rendering it a promising candidate for clinical translation [6], [7], [8], [9]. Our previous work also highlighted the cardioprotective effects of BDMC against staurosporine (STS)-induced cardiomyocyte injury via the PI3K-AKT-Nrf2/HO1 pathway [10], indicating that BDMC has significant therapeutic potential for HFpEF. However, the efficacy and underlying mechanism of BDMC in HFpEF remain to be further investigated.

This study systematically deciphered BDMC’s cardioprotective effects through HFpEF animal model and cell experiments. In vivo, a HFD/L-NAME-induced HFpEF murine model revealed BDMC’s capacity to ameliorate key pathological features: 1) improved diastolic function (E/E’ ratio reduction), 2) attenuated myocardial fibrosis and oxidative stress, and 3) enhanced exercise tolerance, suggesting BDMC exerts direct cardioprotective effects. In vitro, TGF-β induced cardiac fibrosis and oxidative stress in iCFs confirmed the anti-fibrotic and anti-oxidative stress effects of BDMC.

Further, after the in vivo and cell-based experiments, the analysis of therapeutic targets and the docking calculations were performed by integrating proteomics analysis and network pharmacology analysis. Namely, our multi-omics approach of network pharmacology identified 49 potential therapeutic targets enriched in cardiovascular complications such as diabetes and myocardial infarction. Among which, 23 candidate target molecules are annotated to be located in the cell membrane. After molecular docking between BDMC with these 23 targets, the first 10 targets according to the binding energy were chosen for further validation using RT-qPCR. RT-qPCR identified the mRNA expression of six membrane-associated targets were significantly changed in HFpEF mice after BDMC treatment: TGFBR1, IGF1R, NOS3, ESR1, PIK3CG, and GSK3B.

The TGF-β pathway orchestrates cardiac remodeling through fibrosis and apoptosis [27]. While TGFBR1 knockout mitigates post-MI cardiac dysfunction [28], and BDMC modulates TGFB1-mediated extracellular matrix production [29], our study provides novel evidence of BDMC's TGFBR1-targeted activity in HFpEF pathophysiology. IGF-1 signaling modulation, IGF-1’s pleiotropic effects on cardiac inflammation and fibrosis [30] align with our findings of BDMC-mediated IGF1R upregulation. This observation extends previous reports of IGF1R attenuating Ang II-induced fibrosis [31], suggesting that BDMC may activate IGF-1 signaling cascades. NOS3/NO pathway dynamics, NOS3-derived nitric oxide regulates vascular homeostasis and myocardial function [32], [33]. The observed NOS3 suppression corresponds with L-NAME’s pharmacological action, while partial restoration of NOS3 expression by BDMC implies nuanced modulation of NO signaling - a finding of particular interest given the paradoxical effects of NOS isoforms in HFpEF therapy [34]. Estrogen receptor cross-talk, Estrogen receptor α (ESR1) demonstrates cardioprotective effects through anti-apoptotic and anti-inflammatory mechanisms in preclinical models [35], [36]. ESR1 upregulation suggests potential estrogen-mimetic properties, though clinical implications require careful evaluation given unresolved human data [37]. Metabolic regulation via dual role of GSK3B/PI3K-AKT, GSK-3β in cardiomyocyte metabolism and survival [38] converges with insulin-mediated AKT/GSK-3β phosphorylation [39]. Activation of PI3K-AKT signaling, consistent with our prior work on STS-induced injury [10] and endothelial protection findings by Ying et al. [40], reinforces its role in maintaining metabolic homeostasis and cell survival. Collectively, these results indicated that BDMC may exert cardiac protective effects through multiple targets.

In order to screen the potential target of BDMC against HFpEF in further, proteomics analysis was conducted and integrated with the 6 targets mentioned above (1 up-regulated target and 5 down-regulated targets) identified by network pharmacology analysis. The results showed that TGFBR1 was up-regulated in HFpEF compared with Control while down-regulated in HFpEF-BDMC compared with HFpEF (Fig. 5C). Further Western Blotting experiments, molecular docking and molecular dynamics analyses also showed that BDMC binds within the cavity of TGFBR1 (Fig. 6). Furthermore, fibrosis mediated by the TGF-β/SMAD pathway is a key pathological process in HFpEF [25], [26], which can be inhibited by BDMC.

Therefore, BDMC could exert anti-fibrotic effects by inhibiting TGF-β/SMAD pathway, downregulating TGFBR1 and the phosphorylation of TGFBR1 and SMAD2/3, inhibiting the expression of fibrosis related genes, including PCNA, α-SMA and COLA3, resulting in cardioprotective effects. Besides, the anti-oxidant stress effects of BDMC as shown from ATP and ROS detection further contribute to its cardioprotective actions (Figs. 8B, 8C). Mitochondrial ultrastructure obtained by transmission electron microscopy (TEM), immunofluorescence of mitochondrial structure using Mitotracker and mitoSOX (Fig. 8D-8J) indicated the anti-oxidant stress effects of BDMC depend on its effects on mitochondrial. Specifically, BDMC pretreatment reversed the TGF-β-induced downregulation of fusion proteins MFN1 and MFN2 in iCFs, while conversely suppressing the upregulation of the fission protein DRP1. The steady expression of TOMM20 across groups confirms the specificity of these changes (Fig. 8K-8L). Therefore, we conclude that BDMC exerts its anti-oxidative stress action via regulating mitochondrial dynamics.

However, this study has several limitations. First, the sample sizes, while consistent with ethical guidelines and field standards for exploratory research, are relatively small. While statistically significant results and notable effect trends were observed, the limited sample size inherently constrains statistical power and results in wider confidence intervals around effect estimates. Therefore, these findings should be interpreted as preliminary and require validation in future, larger-scale independent studies. Second, a formal a priori power calculation was not performed, as reliable effect size parameters for such exploratory investigations are difficult to pre-define. The effect sizes observed in this study can serve as a valuable reference for designing future confirmatory studies with formal power analysis. Besides, though elucidating novel mechanisms, 3 limitations warrant attention: 1) The paradoxical effects of NOS isoforms may underlie inconsistent clinical outcomes with NO-based therapies [34], necessitating isoform-specific investigations; 2) TGFBR1 as the target of BDMC requires further validation through conditional knockout models. 3) Mechanisms of BDMC on mitochondrial dynamics need further explorations.

## 5 Conclusions

In conclusion, our study demonstrated the significant anti-fibrotic and anti-oxidative stress effects of BDMC against HFpEF. By integrating proteomics analysis and a network pharmacology analysis, TGFBR1 was screened as the potential target of BDMC against HFpEF. Further Western Blotting experiments, molecular docking and molecular dynamics also showed that BDMC exerts cardioprotective effects through regulating TGF-β/SMAD pathway related fibrosis and anti-oxidant stress by moduating mitochondrial dynamics. These findings provide a robust framework for clinical translation while highlighting the need for target-specific validation studies.

## Institutional Review Board Statement

All animal experiment protocols were approved by the Institutional Animal Care and Use Committee of FMMU (protocol number 20220690), and all experiments were performed in accordance with the Guide for the Care and Use of Laboratory Animals published by the National Institutes of Health guide for the care and use of Laboratory animals (NIH Publications No. 8023, revised 1978).

## CRediT authorship contribution statement

**Rong Xu:** Writing – original draft, Methodology, Conceptualization. **Guihua Cao:** Validation, Methodology. **Liming Hou:** Validation, Methodology. **Wei Fu:** Validation. **Xu Li:** Validation. **Chenting Bi:** Data curation. **Xiaoming Wang:** Writing – review & editing, Conceptualization.

## Declaration of Competing Interest

The authors declare that they have no known competing financial interests or personal relationships that could have appeared to influence the work reported in this paper.
