# Supplementary material for: Bisdemethoxycurcumin attenuates myocardial fibrosis in heart failure with preserved ejection fraction by targeting TGFBR1 and oxidative stress
Source: Comput Struct Biotechnol J. 2026 Jan 14;31:422–35. doi: 10.1016/j.csbj.2026.01.009 (PMC12855596; doi:10.1016/j.csbj.2026.01.009)
Supplement: Supplementary Figure S1 — Supplementary material [file mmc1.pdf]

西力生物
BBP BioBioPha

Natural Product Library for Drug Discovery

CERTIFICATE OF ANALYSIS

BBP No.: BBP03060

CAS No.: 52328-96-8

Chemical Name: Bisdemethoxycurcumin

Molecular Formula: $C_{19}H_{16}O_4$

Structure:

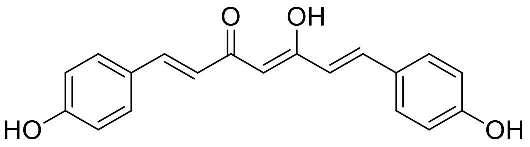

Purity: 98%

Appearance: Orange powder

Solvent: Acetone, methanol

Storage: Store in a dark place under the temperature of −20 °C

Intended Use: For laboratory use only

Reference: K. Inoue, J Agric Food Chem, 2008, 56(20), 9328-9336

Warm Notice: When publishing, please cite as: ***chemical name*** was purchased from BioBioPha Co., Ltd. (Kunming, China)

Characterization Data Summary

Analytical Test	Results
Identification by ^{1}H-NMR	Consistent with the above structure
Purity tested by HPLC, ^{1}H-NMR	98%

Authorized Signature:

Date:

云南西力生物技术股份有限公司
质检部
检验合格
BioBioPha Co., Ltd.

Tel.: +86 871 6521 7109 Fax: +86 871 6521 5563 E-mail: sales@mail.biobiopha.com

西力生物
BBP BioBioPha

Natural Product Library for Drug Discovery

PRODUCT QUALITY REPORT

Product Number: BBP03060

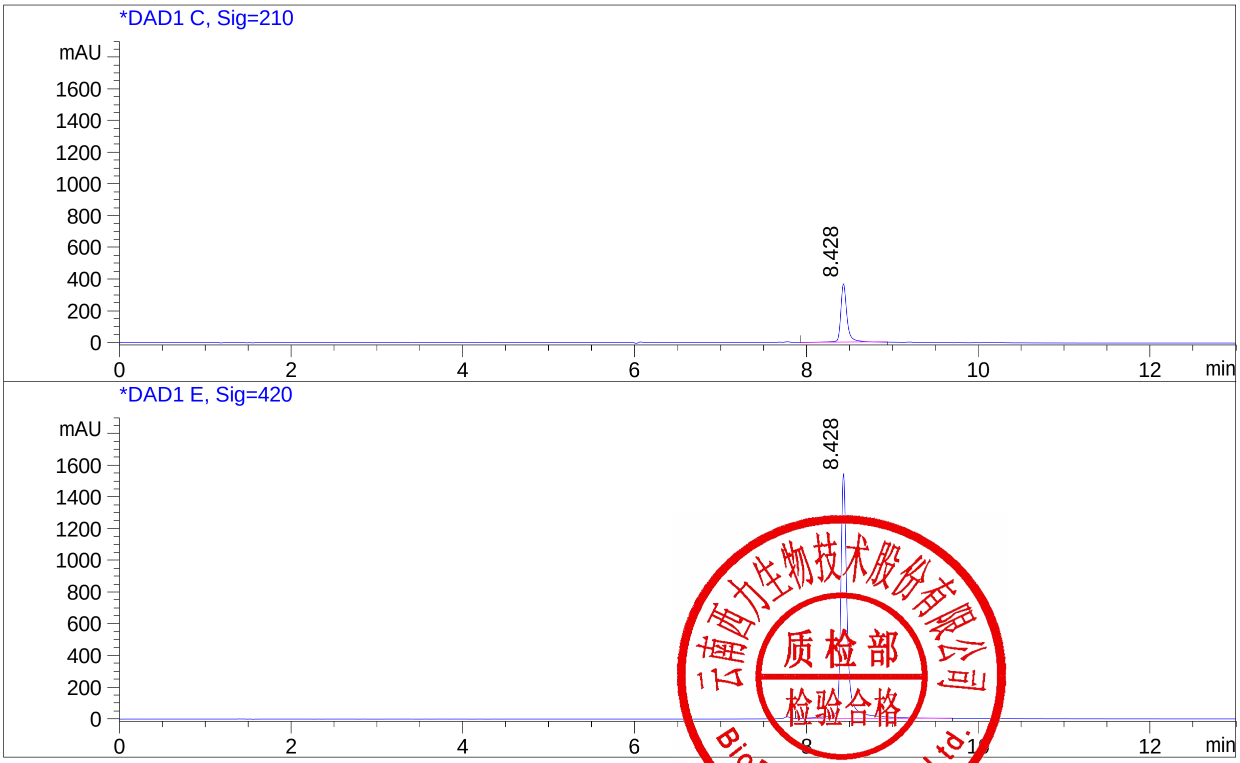

云南西力生物技术股份有限公司
质检部
检验合格
BioBioPha Co., Ltd.

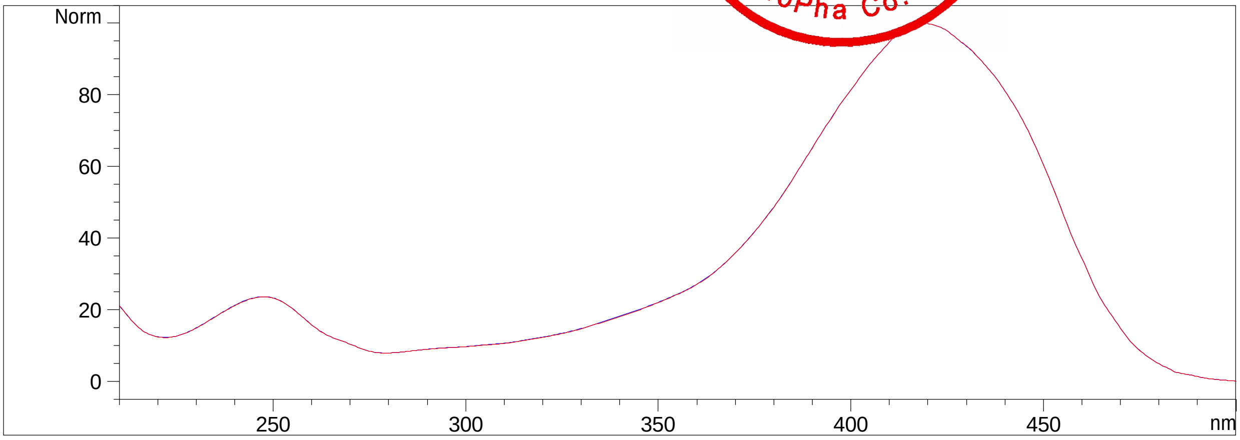

Agilent 1200 series HPLC system
Thermo Hypersil BDS C18 column (5 µm, 4.6 × 150 mm)
20%→100% MeOH in H_2O over 8.0 min followed by 100% MeOH to 13.0 min
1.0 ml/min, 20°C

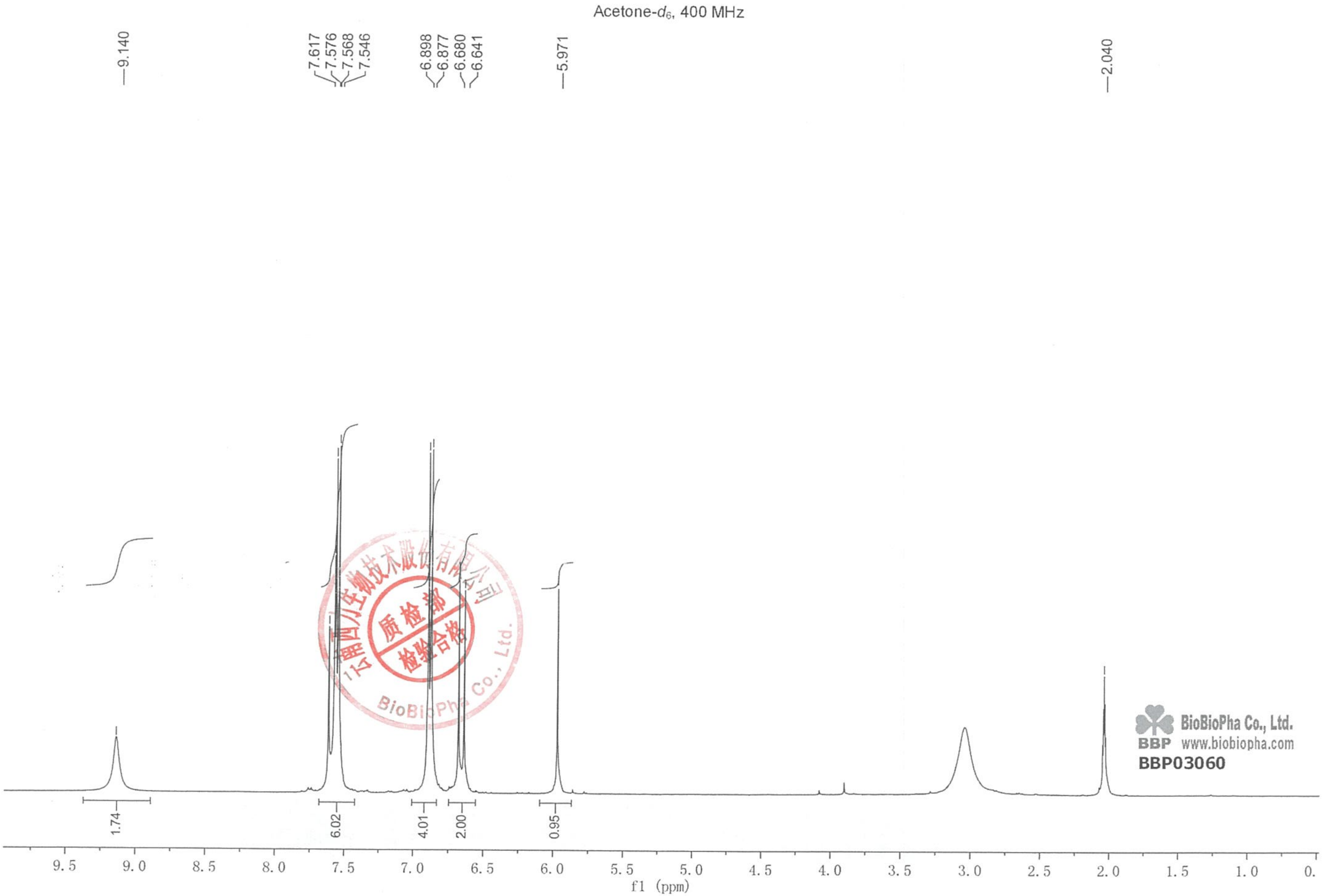
Acetone-d_6, 400 MHz
9.140
7.617
7.576
7.568
7.546
6.898
6.877
6.680
6.641
5.971
2.040
1.74
6.02
4.01
2.00
0.95
9.5 9.0 8.5 8.0 7.5 7.0 6.5 6.0 5.5 5.0 4.5 4.0 3.5 3.0 2.5 2.0 1.5 1.0 0.
f1 (ppm)
BioBioPha Co., Ltd.
BBP www.biobiopha.com
BBP03060
质检部
检验合格
BioBioPha Co., Ltd.
