# Supplementary material for: Bisdemethoxycurcumin attenuates myocardial fibrosis in heart failure with preserved ejection fraction by targeting TGFBR1 and oxidative stress
Source: Comput Struct Biotechnol J. 2026 Jan 14;31:422–35. doi: 10.1016/j.csbj.2026.01.009 (PMC12855596; doi:10.1016/j.csbj.2026.01.009)
Supplement: Supplementary Figure S2 — Supplementary material [file mmc2.doc]

**Supporting information**

**Bisdemethoxycurcumin attenuates myocardial fibrosis in Heart Failure with Preserved Ejection Fraction by targeting TGFBR1 and oxidative stress**

Rong Xu, Guihua Cao, Liming Hou, Wei Fu, Chenting Bi, Xu Li, Xiaoming Wang*

Department of Geriatrics, Xijing Hospital, The Airforce Military Medical University, Xi’an, Shaanxi 710032, China

*Correspondence: xmwang@fmmu.edu.cn; Tel.: 86-29-84771818, Department of Geriatrics, Xijing Hospital, The Airforce Military Medical University, Xi’an, Shaanxi 710032, China.

**Supplementary Figure S1**


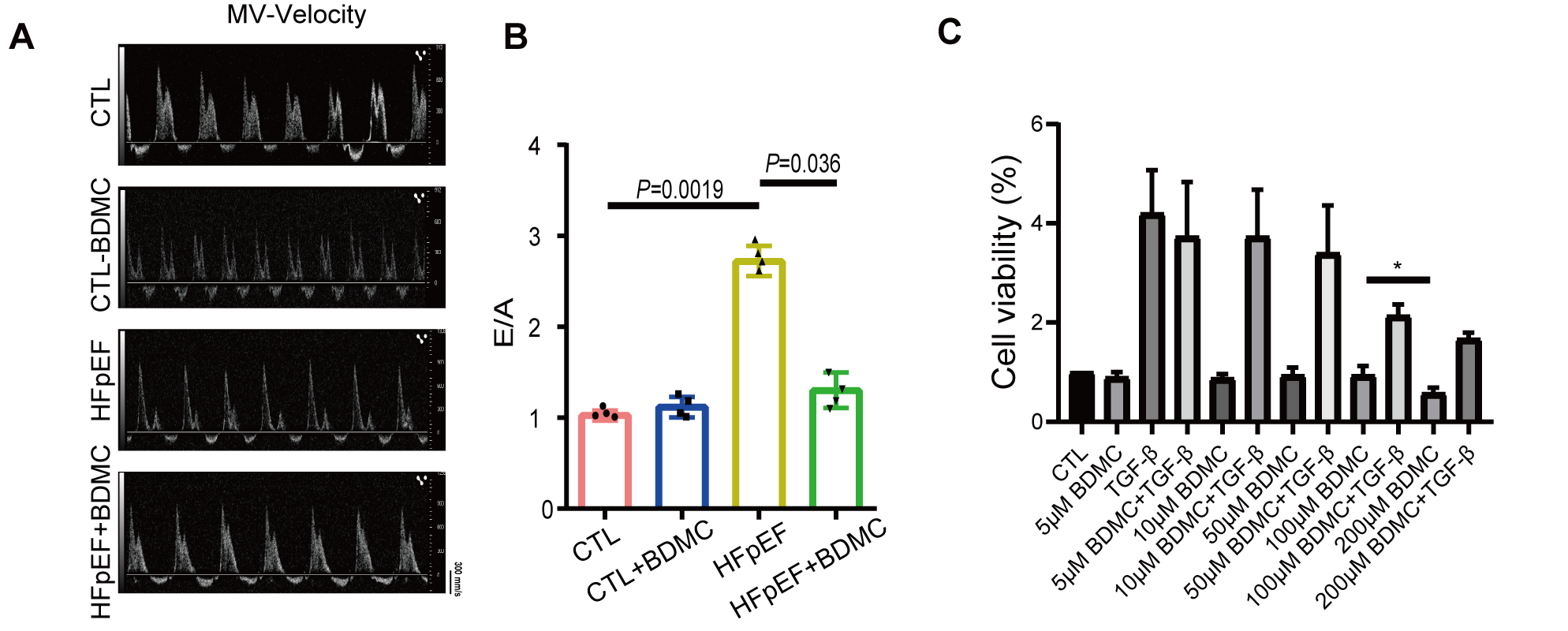


**Figure S1. A-B.** Impact of BDMC on cardiac E/A. n=6. **C.** Cell viability of indicated groups. The optimum concentration of BDMC was searched in different concentration gradient from 5μM to 200μM in TGF-β induced iCFs cells. The optimum concentration of BDMC is 100 μM.

**Supplementary Table S1**

| Gene Names | Abundance Ratio: (HFpEF) / (CTL) | Abundance Ratio Adj. P-Value: (HFpEF) / (CTL) | Abundance Ratio: (HFpEF-BDMC) / (HFpEF) | Abundance Ratio Adj. P-Value:(HFpEF-BDMC) / (HFpEF) |
| --- | --- | --- | --- | --- |
| TGFBR1 | 1.752 | 0.047071767790197 | 0.187637969 | 0.0399418532864673 |
| NOS3 | 0.01 | 2.60076481835564E-16 | 0.414634146341463 | 0.419327819476702 |
| PIK3CG | 1.207 | 0.956201748034333 | 1.30635838150289 | 0.60156003458472 |
| GSK3B | 0.647 | 0.690950956136508 | 0.760849492151431 | 0.536408842486969 |
